# Effects of a multicomponent communication training to involve older people in decisions to DEPRESCRIBE cardiometabolic medication in primary care (CO-DEPRESCRIBE): protocol for a cluster randomized controlled trial with embedded process and economic evaluation

**DOI:** 10.1186/s12875-024-02465-7

**Published:** 2024-06-11

**Authors:** Peter J.C. Stuijt, Mette Heringa, Liset van Dijk, Adrianne Faber, Jako S. Burgers, Talitha L. Feenstra, Katja Taxis, Petra Denig

**Affiliations:** 1 Department of Clinical Pharmacy and Pharmacology, University of Groningen, University Medical Centre Groningen, PO-Box 30001, HPC AP50, UMCG, Groningen, 9700RB The Netherlands; 2 SIR Institute for Pharmacy Practice and Policy, Leiden, The Netherlands; 3 Nivel, Netherlands Institute for Health Services Research, Utrecht, The Netherlands; 4 Unit of PharmacoTherapy, - Epidemiology and -Economics, Groningen Research Institute of Pharmacy, University of Groningen, Groningen, The Netherlands; 5 Department of Family Medicine, School CAPHRI, Maastricht University, Maastricht, The Netherlands; 6 Dutch College of General Practitioners, Utrecht, The Netherlands; 7 Dutch National Institute of Public Health and the Environment (RIVM), Bilthoven, The Netherlands

**Keywords:** Deprescribing, Shared decision making, Patient-centered care, Primary care, Barriers- facilitators, Provider-patient communication

## Abstract

**Background:**

Deprescribing of medication for cardiovascular risk factors and diabetes has been incorporated in clinical guidelines but proves to be difficult to implement in primary care. Training of healthcare providers is needed to enhance deprescribing in eligible patients. This study will examine the effects of a blended training program aimed at initiating and conducting constructive deprescribing consultations with patients.

**Methods:**

A cluster-randomized trial will be conducted in which local pharmacy-general practice teams in the Netherlands will be randomized to conducting clinical medication reviews with patients as usual (control) or after receiving the CO-DEPRESCRIBE training program (intervention). People of 75 years and older using specific cardiometabolic medication (diabetes drugs, antihypertensives, statins) and eligible for a medication review will be included. The CO-DEPRESCRIBE intervention is based on previous work and applies models for patient-centered communication and shared decision making. It consists of 5 training modules with supportive tools. The primary outcome is the percentage of patients with at least 1 cardiometabolic medication deintensified. Secondary outcomes include patient involvement in decision making, healthcare provider communication skills, health/medication-related outcomes, attitudes towards deprescribing, medication regimen complexity and health-related quality of life. Additional safety and cost parameters will be collected. It is estimated that 167 patients per study arm are needed in the final intention-to-treat analysis using a mixed effects model. Taking loss to follow-up into account, 40 teams are asked to recruit 10 patients each. A baseline and 6-months follow-up assessment, a process evaluation, and a cost-effectiveness analysis will be conducted.

**Discussion:**

The hypothesis is that the training program will lead to more proactive and patient-centered deprescribing of cardiometabolic medication. By a comprehensive evaluation, an increase in knowledge needed for sustainable implementation of deprescribing in primary care is expected.

**Trial registration:**

The study is registered at ClinicalTrials.gov (identifier: NCT05507177).

**Supplementary Information:**

The online version contains supplementary material available at 10.1186/s12875-024-02465-7.

## Background

For frail older patients, the potential long-term benefits of intensive cardiometabolic treatment can be outweighed by increasing risks of adverse effects and medication burden. In those cases deprescribing could be considered [1, 2]. Deprescribing can be defined as the process of stopping or reducing medication, supervised by a healthcare provider (HCP), with the goal of managing polypharmacy, reducing drug-related problems and improving patient outcomes [3, 4]. Deprescribing of medication for cardiovascular risk factors and diabetes has been incorporated in several clinical primary care guidelines, but proves to be difficult to implement [1, 2, 5–7]. Observational studies illustrate that deprescribing of cardiovascular or diabetes medication may not occur in 3 out of 4 patients who are eligible for less strict medication treatment [8–10], potentially resulting in excessive medication burden and preventable adverse events. Several barriers to specifically deprescribing cardiometabolic have been identified [11–13]. For patients, the idea of deintensifying medication they have taken for a long time and allowing less strict target values than before can be disturbing or confusing [11, 12, 14]. HCPs may perceive uncertainty and a lack of evidence about the potential benefits and risks of stopping cardiometabolic medication [11, 13]. In addition, they can experience a lack of communication skills and tools to involve older and frail patients in discussing potential benefits and risks [13].

Identifying medication for cessation, partnership with patient and carers, planning the deprescribing regimen and monitoring are seen as core to the deprescribing process [15]. In Dutch primary care, clinical medication reviews (CMRs) are routinely conducted jointly by the general practitioner and the community pharmacist, who are responsible for the management and monitoring of long term health conditions and associated pharmaceutical care, respectively. Randomized controlled trials have shown the potential of CMRs to effectively reduce the number of drugs prescribed [16–20]. As such, deprescribing can be integrated in CMRs [21]. Elements that may contribute to the success of CMRs in terms of deprescribing are the involvement of local multidisciplinary healthcare teams including a pharmacist, having clearly defined roles, preparing patients in advance for discussing risks and benefits of deprescribing, and ensuring to have appropriate follow-up plans [22, 23].

Deprescribing is not yet included in routine education and training of HCPs [24]. Additional training programs are needed for successful implementation of deprescribing cardiometabolic medication in primary care settings [9, 25]. Given the potential of CMRs and the importance of a multidisciplinary approach, training of HCP teams that conduct CMRs is pertinent [24]. Based on the barriers perceived by HCPs, the training should provide information on available evidence regarding benefits and risks as well as tools and guidance on discussing possibilities for deprescribing cardiometabolic medication with patients. Previously, a pharmacist-led intervention was developed and tested that was aimed at deprescribing cardiometabolic medication in patients with type 2 diabetes with a high hypoglycemia risk [26, 27]. For the intervention, pharmacists conducted a tailored CMR after a 6-hour training on deprescribing cardiometabolic medication using evidence-based guidelines, medication management to prevent adverse events, and skills for conducting patient consultations about deprescribing. This intervention led to increased deprescribing of cardiometabolic medication [26]. The process evaluation indicated that the intervention could be improved to include particularly patients more likely in need of deintensifying cardiometabolic medication [27]. Furthermore, extending the training on communication skills and how to discuss deprescribing with patients who do not experience adverse effects of their medication could enhance the implementation of deprescribing [27].

Building on these findings, a blended training program with supportive tools was developed, named “Communication training to involve Older people in decisions to DEPRESCRIBE cardiometabolic medication in primary care (CO-DEPRESCRIBE)” (see Additional file 1). The training program focuses on patient-centered communication and shared decision making in the context of CMRs, and aims to enable HCPs to have constructive conversations with patients of 75 years and older about deprescribing of their cardiometabolic medication. Additional components pay attention to content and organizational aspects related to barriers and facilitators relevant for the implementation of deprescribing such medication. This paper describes the protocol of the CO-DEPRESCRIBE study, including results of a small pilot study to test the feasibility of patient recruitment and data collection procedures.

## Objectives

The overall aim of the CO-DEPRESCRIBE study is to evaluate a multicomponent communication training program for multidisciplinary HCP teams about deprescribing of cardiometabolic medication in older patients in Dutch primary care. Specific objectives are to evaluate:

1. the effects of the CO-DEPRESCRIBE intervention on changes in medication, patient-reported outcomes, and clinical outcomes.
2. the effects of the intervention on patient-centered communication and shared decision making during CMR patient consultations.
3. the process and feasibility of implementing the intervention in Dutch primary care.
4. the cost-effectiveness of the intervention.

## Methods

### Study design

**Fig. 1:**
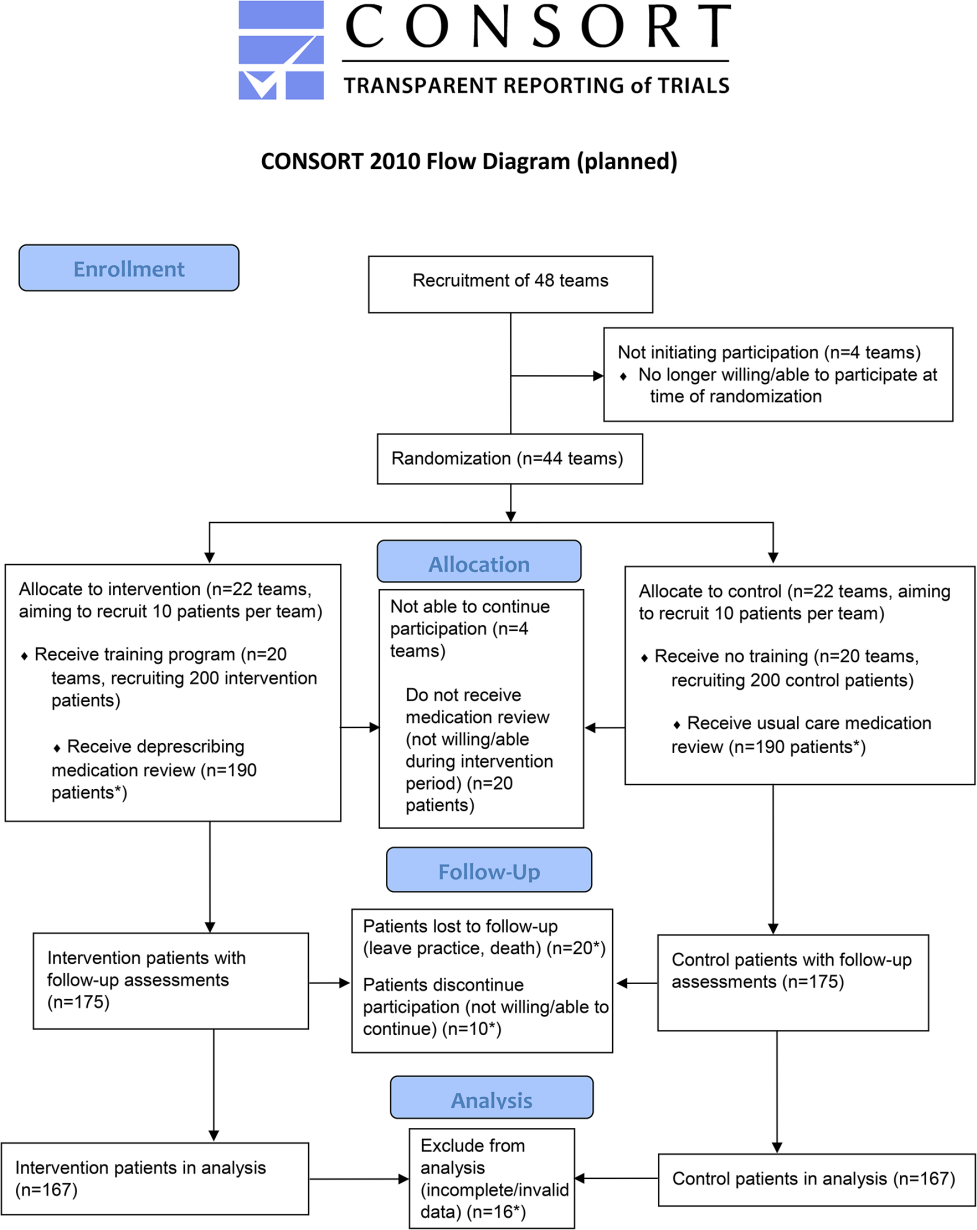
CONSORT Flow diagram. * Numbers of expected loss of teams and patients are based on previous practice-oriented research in a similar population in the Netherlands [26]

A pragmatic parallel two-arm cluster-randomized controlled trial will be conducted in Dutch primary care. Additionally, a mixed method process evaluation and a cost effectiveness study alongside this RCT will be conducted. HCP teams are 1:1 allocated to the intervention group receiving the training before data collection, or the control group receiving the training after data collection (Fig. 1). Teams in the intervention group will conduct CMRs focused on deprescribing cardiometabolic medication, while teams in the control group conduct regular CMRs (care as usual). HCP teams are the unit of randomization for the intervention, while outcomes are measured at patient level.

### Setting

The intervention approach aligns with how CMRs are routinely performed in the Dutch primary care, where local teams of community pharmacists and general practitioners conduct CMRs together. Locally, most community pharmacists work together with general practitioners in so-called Pharmaceutical Therapy Audit Meeting groups (‘FTOs’ in Dutch). In such groups, local agreements can be made about organizing pharmaceutical care. Pharmacists and general practitioners often have agreements on how to conduct CMRs and the division of roles. In some rural areas without community pharmacists, dispensing general practitioners may conduct CMRs on their own, but in most areas pharmacists are usually in the lead for conducting CMRs. Other HCPs, such as pharmacy consultants and nurse practitioners, may be involved in patient consultations or follow-up. CMRs are recommended and usually reimbursed by health insurers for people of 75 years and older using 10 or more medications for longer than 3 months and/or established frailty.

The CO-DEPRESCRIBE intervention is developed to align with both current practices in conducting CMRs and the Dutch guideline [28], which recommends:

1. a(n initial) consultation with the patient to collect information on the patient’s complaints, concerns, expectations and experiences with the current medication use.
2. a pharmacotherapeutic analysis to identify potential drug-related problems, including the use of explicit criteria for stopping or starting medication.
3. drafting a treatment plan by the community pharmacist together with the general practitioner (GP), preferably in a joint meeting, and, if necessary, after consulting medical specialists involved in the medication treatment.
4. a (follow-up) consultation to discuss and finalize the treatment plan with the patient.
5. organizing follow-up and monitoring according to agreements made between the community pharmacist, the GP and the patient.

**Fig. 2:**
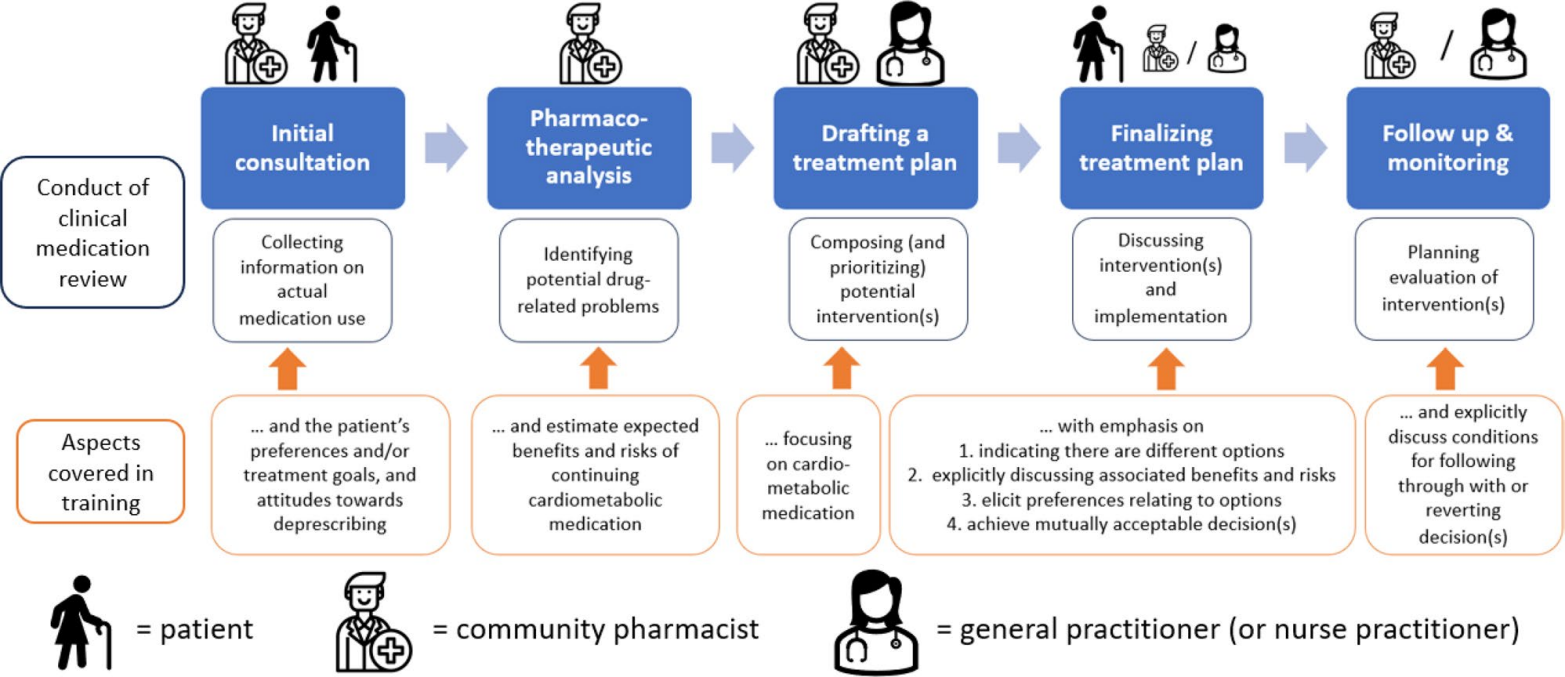
Conduct of clinical medication reviews: who is involved and what are CO-DEPRESCRIBE training aspects

The CO-DEPRESCRIBE specific aspects added to this generic CMR approach are detailed in Fig. 2.

### Study populations

An HCP team is eligible for participation if it consists of:

- ≥ 1 community pharmacist(s) and ≥ 1 GP(s), who are involved in conducting CMRs for shared patients registered in the pharmacy and general practice, or
- ≥ 1 dispensing GP(s) who is involved in conducting CMRs, where the HCP(s) from these practices who will initiate conversations with patients as part of the CMRs intend(s) to follow the CO-DEPRESCRIBE training. A pharmacy or general practice that participates in another randomized trial focusing on deprescribing is excluded from participation.

Patients are eligible for participation, if:

- ≥ 75 years of age.
- exposed to polypharmacy, operationalized as having received at least 3 dispensings for at least 5 different medications in the previous year.
- stable use (operationalized as having received at least 2 dispensings in the first 8 months and at least 1 dispensing in the last 4 months of the previous year) of 1 or more of the following cardiometabolic medications:

- sulfonylurea derivative.
- insulin.
- any 2 glucose-lowering medications (see medications listed in ‘Outcome and other parameters’ section).
- any 2 blood pressure-lowering medications (see medications listed in ‘Outcome and other parameters’ section).
- statin.

Patients are excluded if they are diagnosed with type 1 diabetes, have already received a CMR in the previous 12 months, do not understand the Dutch language or are not able to give informed consent.

### Recruitment and allocation

For the recruitment of HCP teams, the Dutch Association of Pharmacists and regional primary care organizations are requested to feature information about the study in their newsletters and on social media platforms. Simultaneously, targeted telephone outreach to pharmacists are conducted to gauge interest and provide information directly. After informed consent is obtained from a representative of the local HCP team, the team is allocated to either the intervention or control group. A fixed block randomization with a block size of 4 is performed by a staff member of the University Medical Center Groningen’s research support department using ‘ALEA Datamanagement’ software version 18.5. A stratum for team type with 2 categories is used for teams led by a community pharmacist and teams led by a dispensing general practitioner, respectively. Given the nature of the study, it is not possible to blind participants.

For participating teams in both arms, patients eligible based on age, polypharmacy and cardiometabolic medication use are identified by the researchers based on routine data available at the pharmacy. HCPs are asked to apply the exclusion criteria and invite eligible patients to participate in the study and ask for permission to share contact details with the researchers. Once they grant permission, the researchers inform patients about the study and obtain their informed consent to minimize the burden for HCP teams. Each HCP team is requested to keep inviting eligible patients until at least 10 patients have given consent to participate.

### CO-DEPRESCRIBE intervention

The CO-DEPRESCRIBE intervention was developed jointly by experts from Nivel (Netherlands institute for health services research), the SIR Institute for Pharmacy Practice and Policy, the University of Groningen and the University Medical Center Groningen. It builds on previous research and incorporates aspects of the Calgary Cambridge consultation model [29], shared decision making models [30, 31], barriers and facilitators to deprescribing, including patient attitudes and typologies [32] as well as content and organizational aspects regarding deprescribing [1]. The blended training consists of 5 modules: e-learning including short lectures with questions and self-assessment tasks (2 modules), half-day face-to-face group training sessions (2 modules) and an online communication training including personal feedback on a videotaped patient consultation (1 module) [33] (Table 1). A detailed description of the training modules can be found in Additional file 2. The group sessions are delivered by 2 trainers (AF and AMKD), who are pharmacist-educators with 10 years’ experience in providing postgraduate training for community pharmacists and multidisciplinary HCP teams.

**Table 1:** An overview of the CO-DEPRESCRIBE training program

Module	Focus	Description of content	Method	Time investment
Module 1 e-learning	Dutch multidisciplinary guideline module for “Deprescribing medication”	Introducing the process deprescribing: the process, goal, and why it is important. Also: suitable patients and moments for deprescribing, medication-specific protocols for deprescribing, tools for weighing possibilities and involving patients in the decision- making process	Short web lectures with questions and self-assessment tasks	± 1.5 h
Module 2 e-learning	How to talk about possibilities for deprescribing	Patient-centered communication, collecting information on the patient’s preferences and attitudes regarding deprescribing, and key elements of shared decision making in clinical practice	Short web lectures with questions and self-assessment tasks	± 1 h
Module 3 face-to-face group training	Applying deprescribing guidelines in practice	Medication-specific deprescribing guidelines and complex patient cases	Quiz, short presentations and group-based exercises	± 2 h
	Collecting information on the patient’s personal preferences, goals, attitudes and experiences	Practicing collecting information on a patient’s personal preferences and attitudes regarding cardiometabolic treatment during a patient consultation about possibilities for deprescribing and following the Calgary-Cambridge model	Short presentations and pair-based exercises, including role play	± 2 h
Module 4 face-to-face group training	Applying risk tools and addressing experienced barriers and facilitators	Practicing with the application of risk calculators (e.g., U-Prevent and HASBLED) for weighing potential benefits and risks. Also, reflecting on trainees’ experienced barriers and facilitators in daily practice	Short presentations and group-based exercises	± 2 h
	Applying shared decision making in the context of deprescribing consultations	Information on why shared decision making is important, the different steps of shared decision making, applying reflective listening and practicing consultation skills during mock patient consultations	Short presentations, pair-based exercise and a group-based exercise, including role-play with a professional training actor simulating the patient role	± 2 h
Module 5 online feedback	Applying trained elements of communication and shared decision making in practice	Applying all consultation aspects in a clinical medication review focusing on deprescribing conducted in daily practice with reflection and feedback on five domains.	Performing self-reflection and receiving feedback from trainer on video-recorded consultations	± 1 h

Supportive tools are provided as part of the CO-DEPRESCRIBE intervention and also used during the training (Table 2).

**Table 2:** Supportive tools for the CO-DEPRESCRIBE intervention

Tool(s)/instrument(s)	Purpose	Content
Patient leaflet	Preparing patients for discussing possibilities to deprescribe	The leaflet explains that some patients can benefit from deprescribing, and stimulates the patient to think about what is important for them regarding their cardiometabolic treatment
Graphical placemat	Navigating a conversation about possibilities for deprescribing cardiometabolic medication	This placemat depicts potentially important themes and subsequent questions to discuss, and can be used by the HCP and the patient for prioritizing topics
Conversation aid #1: collecting personal information	Eliciting a patient’s personal preferences, attitudes, concerns and/or experiences related to deprescribing cardiometabolic medication	This conversation aid contains example questions that an HCP can use for different topics
Conversation aid #2: for shared decision making [31]	Following the 4 steps of shared decision making	This conversation aid summarizes the 4 steps, and provides exemplary phrases for each step.
Outcome Prioritization Tool (OPT) [34]	Eliciting a patient’s prioritization of treatment goals	The OPT invites patients to indicate with a slider from 0 to 100 how important “keeping you alive”, “maintaining independence”, “reducing pain” and “reducing other symptoms” are for them personally.
U-Prevent, CHA2DS2–VASc [35], HASBLED [36] and ORBIT [37] risk calculators	Risk calculations can help the HCP to explain and/or weigh potential benefits and risks of continuing or discontinuing medication.	Risk calculations estimate probabilities of survival without myocardial infarction, stroke or bleeding in relation to cardiovascular risk factor values. Several U-Prevent calculators also show how using cardiometabolic medication and/or achieving different clinical target levels affect these estimates.

After receiving the training, HCPs should be able to:

- identify patients likely to benefit from deprescribing cardiometabolic medication.
- know and apply the drug-specific recommendations for deprescribing cardiometabolic medication as provided in the national guideline module “Deprescribing medication”[1].
- identify barriers and facilitators for deprescribing cardiometabolic medication from the perspective of the patient.
- describe and implement the conditions and working processes needed for deprescribing of cardiometabolic medication including establishing modes for inter-professional communication.
- apply key-elements of shared decision making in consultations with patients about deprescribing.
- apply techniques that enhance patient-centered communication in order to identify and acknowledge a patient’s attitudes towards deprescribing.

### Outcome measures and other parameters

The primary outcome is the percentage of patients with deprescribing of cardiometabolic medication, that is, at least 1 cardiometabolic medication deintensified within a half year after the initial consultation conducted as part of the CMR. Deintensification is defined as either a decreased dosage (for all medication except insulin) or dosing schedule (for insulin) or a discontinuation of medication (Table 3). Medications of the following therapeutic groups and pharmacological subgroups are included: glucose-lowering medication (Anatomical Therapeutic Chemical code A10: insulins, biguanides, sulfonylureas, alpha glucosidase inhibitors, thiazolidinediones, dipeptidyl peptidase 4 inhibitors, glucagon-like peptide 1 analogues, sodium glucose co-transporter 2 inhibitors, other glucose-lowering drugs), blood pressure-lowering medication (Anatomical Therapeutic Chemical codes C03: diuretics excluding C03DA04/05 and C03XA01, C07: beta blocking agents, C08: calcium channel blockers, C09: renin-angiotensin-system inhibitors, C02: other antihypertensives), statins (Anatomical Therapeutic Chemical code C10AA), and antithrombotic agents (Anatomical Therapeutic Chemical code B01, excluding B01AB, B01AD and B01AX) [38]. Changes in dosages will first be assessed at chemical substance level and subsequently grouped at subgroup level to account for simultaneous dosage decreases and increases at subgroup level. Discontinuations are also defined at subgroup level to account for medication switching at this level. Switches to less potent and lower risk medication in accordance to deprescribing guidelines are considered deintensifications. In case of missing dosage information, medication is considered deintensified when the strength of the follow-up dispensing is lower than that of the baseline dispensing.

**Table 3:** Definitions for assessing cardiometabolic medication deintensification

Medication use	≥ 1 dispensing in 120 days up to initial consultation (baseline use) ≥ 1 dispensing within 120-day time window around 182 days after initial consultation (follow-up use)
Decrease in dosage	Daily dose for last dispensing up to initial consultation (baseline) is higher than daily dose for dispensing closest to 182 days after initial consultation (follow-up)
Decrease in scheme	Insulin scheme for distinct insulin dispensings in 120 days up to initial consultation (baseline) is more intensive than insulin scheme for distinct insulin dispensings within 120-day time window around 182 days after initial consultation (follow-up)
Discontinuation	≥ 1 dispensing in 120 days up to initial consultation 0 dispensing within 120-day time window around 182 days after initial consultation

To evaluate the effects of the intervention on patient-reported and clinical outcomes, the following secondary outcomes are included:

1. changes in patient-reported health/medication-related complaints with impact [39].
2. changes in patient-reported attitudes towards deprescribing cardiometabolic medications, using the subscales ‘Appropriateness’ and ‘Concerns’ of the revised Patient Attitudes towards Deprescribing questionnaire (rPATD) with a small adaptation to allow for before-after data collection [32, 40].
3. changes in (general) patient-reported attitudes towards deprescribing, using subscales ‘Burden’ and ‘Involvement’ of the rPATD [40].
4. changes in medication regimen complexity, using a simplified Medication Regimen Complexity Index (MRCI) based on availability of dispensing information [41, 42].
5. changes in clinical outcomes: glycated hemoglobin A1c level, systolic and diastolic blood pressure level, low-density-lipoprotein cholesterol level, and cardiovascular disease diagnoses.

To evaluate the effects on communication and shared decision making, the following secondary outcomes are included:

6. patient-reported involvement in the decision making process, using the Shared Decision Making Questionnaire (SDM-Q-9) [43].
7. patient-reported views on HCP communication skills, using the Communication Assessment Tool for Pharmacists (CAT-Pharm) [44].
8. patient-reported experience regarding the CMR focused on deprescribing cardiometabolic medication, based on the patient-reported experience measure (PREM) used in our previous study on deprescribing cardiometabolic medication in patients with type 2 diabetes with a high hypoglycemia [26]. This outcome is also used for the process evaluation.

In addition to the costs related to development and delivery of the intervention, the following secondary outcomes are included to evaluate the cost-effectiveness of the intervention:

9. changes in health-related quality of life (EQ-5D-5L) [45].
10. healthcare consumption, based on the Medical Consumption Questionnaire (iMTA MCQ) [46], using only those items relevant for the current intervention, to avoid unnecessary burden for participants.

Additionally, demographics (age, sex and living situation), frailty based on the Tilburg Frailty Indicator [47] (TFI), health- and medication literacy based on Dutch version of the Set of Brief Screening Questions [48] with adaptations from the multimorbidity treatment burden questionnaire [49] and the RALPH conversation guide [50], body mass index, and cardiovascular disease diagnoses (see data collection) are reported to describe the population. The following clinical parameters are included to estimate a need for deprescribing in accordance with the Dutch guideline [1], to monitor changes in risk factor levels, and to perform subgroup analyses: baseline (that is, before initial consultation) glycated hemoglobin A1c level, systolic and diastolic blood pressure level, low-density-lipoprotein cholesterol level, estimated Glomerular Filtration Rate level. Additionally, data on unplanned hospitalisations and emergency visits will be summarized to explore potential negative effects of the intervention.

### Data collection

The following information of participating HCP teams will be collected at baseline using a structured data collection form: professional discipline, years of experience, relevant professional training received, estimated total number of patients, and number of CMRs conducted last year.

Information on medication dispensings, including the date of dispensing, ATC code, product name including strength, total number of units dispensed, and dosing schedule for included patients from 180 days before the initial consultation to 240 days after the initial consultation will be extracted from the pharmacy information system.

Patient-reported outcome measures are collected at different time points (Additional file 3). For some questionnaires, the preferred mode of administration is verbal (i.e., by telephone interview), to enable clarifications when needed. Otherwise, patients can choose whether they want to complete the questionnaires on paper (send through regular mail), online (link via email) or verbally (telephone interview). When hearing or speech is impaired, telephone interviews can be replaced by paper-based or digital questionnaires.

All patient-reported data and data related to the conduct of the CMRs (see ‘Process evaluation) are collected and managed using REDCap electronic data capture tools hosted at the University Medical Center Groningen [51, 52]. REDCap is a secure, web-based software platform designed to support data capture for research studies, providing 1) an intuitive interface for validated data capture; 2) audit trails for tracking data manipulation and export procedures; 3) automated export procedures for seamless data downloads to common statistical packages; and 4) procedures for data integration and interoperability with external sources.

Clinical data for baseline and follow-up will be collected from general practices’ patient electronic health records. For this, general practices are contacted around 1 year after the initial consultation of the last included patient to collect (as available): glycated hemoglobin A1c level, systolic and diastolic blood pressure level, low-density-lipoprotein cholesterol level, estimated Glomerular Filtration Rate level, body mass index and cardiovascular diseases (International Classification of Primary Care codes K74: angina pectoris, K75/K76: ischemic heart disease, K77: heart failure, K78-K84: other heart diseases, K85-K87: hypertension, K88: orthostatic hypotension, K89: trans ischemic attack, K90: cerebrovascular accident, K91: arthrosclerosis) [53]. For the baseline, the most recent values in a 1-year period prior to the initial consultation are included. For the follow-up, values closest to 182 days after the initial consultation are included. In addition, data on unplanned hospitalisations and emergency visits during the follow-up period are collected.

### Feasibility pilot

To test the feasibility of the proposed procedures for patient recruitment and collecting patient reported data, a small pilot study was conducted. The pharmacists of 3 different pharmacies were asked select patients ≥ 75 years, eligible for a CMR and using 1 or more cardiometabolic medications, and invite them for “a study involving a clinical medication review focusing on possibilities to deintensify cardiometabolic medication”. The aim was to include 3 patients per pharmacist.

Patients consenting to participate were offered the CMR by the pharmacist and received questionnaires from the research team at 5 timepoints via telephone, e-mail or mail. The follow-up questionnaires were administered 6 weeks after the initial consultation, instead of after 6 months as planned for the final study, as the aim was to assess the feasibility of the procedure rather than collecting valid data. Issues resulting in loss of patients, missing data and time needed for data collection were documented.

Two pharmacists invited 8 eligible patients via telephone. Of these patients, 2 were not interested, 2 perceived participating as too burdensome and 1 decided against participation after talking to the nurse practitioner in general practice, who had not been informed about the study, and 3 were willing to participate. Due to time constraints, the 3rd pharmacist asked 6 eligible patients via mail to contact the researcher, which only 1 did, who was willing to participate. This resulted in 4 patients consenting to participate (all males, median age = 79.5 years). All 4 completed the interviews and questionnaires. The average time needed for administration all questionnaires via telephone (*n* = 14) was 26 min, ranging from 16 to 39 min.

Based on these results, it was decided to support HCPs for the recruitment of patients by selecting all eligible patients during an initiation visit and provide a paper-based instruction with how to invite patients by telephone instead of via mail. Moreover, our estimations of the time needed for the different questionnaire administrations in the patient information leaflet were adjusted based on this pilot.

### Sample size

In the previous study, cardiometabolic medication had been deintensified in 48% of the intervention versus 31% of the control patients with type 2 diabetes [26], which constitutes a 17% difference attributable to the training. Expecting that deprescribing has increased due to the introduction of new guidance and increased attention, a deintensification percentage of 36% in the control group is assumed. As the training has been refined and extended in comparison to the intervention tested previously, at least a similar increase in deprescribing is expected in the intervention group. To detect an absolute difference of at least 17% in deintensification between intervention- and control group, 133 patients per study arm are needed (sample size estimation for comparing 2 proportions, 2-sided with a power of 80% and 5% significance level). With a clustering effect of 1.27 (expected intraclass correlation coefficient of up to 0.03 and 10 patients per cluster) 169 patients are needed per study group in the final analyses. With an estimated 15% patient loss to follow-up, 200 patients per study group need to be included. It is expected that each HCP team is able to recruit 10 patients who give informed consent, requiring 20 teams per study group. With an estimated 10% HCP team loss to follow-up, 44 teams need to be randomized (Fig. 1).

### Data analysis

Descriptive baseline characteristics will be reported at HCP and patient level: means and standard deviation or median and interquartile range with number of valid observations, depending on normality of data for continuous data; frequencies and percentages for categorical data. Possible imbalances in patient characteristics between group I and II will be explored with t-tests, Chi^2^ tests or Mann-Whitney U tests. Other parameters will be described for patients per group in similar fashion as the baseline characteristics. Where relevant, minimum and maximum values will be provided.

The effect of the training on the primary outcome, that is, the percentage of patients with deintensification of cardiometabolic medication, will be tested in a logistic mixed effects model including the HCP team as random effect (intention-to-treat analysis). If needed, imbalances in parameters used for describing the population will be controlled for by adjustment in a secondary analysis.

The analysis will be repeated including only patients that received the CMR as intended (per-protocol analysis). Explorative subgroup analyses will be performed focusing on potential differences between patients of both groups in age, sex, frailty, education, living situation, disease history, and estimated need for deprescribing. Additional explorative analyses will be conducted differentiating the outcome per therapeutic class: (a) glucose-lowering medication, (b) blood pressure-lowering medication, (c) statins and (d) antithrombotic agents.

For the unplanned hospitalizations and emergency visits, data from the electronic health records in general practice will be combined with self-reported data on healthcare consumption in the last 3 months. A composite score will be used to distinguish patients with unplanned hospitalizations and/or emergency visits from those without. A difference in composite score between the study groups will be compared with a Chi^2^ test.

The effects of the intervention on secondary outcomes will be compared at patient level:

1. shared-decision making sum score: t-test or Mann-Whitney U test.
2. CAT-Pharm sum score (or percentage of excellent scores, in case of a ceiling effect): t-test or Mann-Whitney U test (or a Chi^2^ test when using percentage of excellent scores).
3. change in number of health/medication related complaints with impact: mixed design ANOVA.
4. change in attitudes towards deprescribing per subscale: mixed design ANOVA.
5. change in medication regimen complexity score, including only the aforementioned cardiometabolic medication: mixed design ANOVA.
6. change in health-related quality of life sum score: mixed design ANOVA.
7. change in the clinical parameters (per parameter): mixed design ANOVA.

When patients completed only part of a questionnaire, missing data will be dealt with following recommendations as provided by the questionnaire developers. For the economic evaluation multiple imputation will be used to account for missing data (see also Additional file 4). For all hypothesis testing, a 2-sided p-value of less than 0.05 will indicate statistical significance.

### Process evaluation

To investigate the feasibility of implementing the intervention on a nation-wide scale in Dutch primary care, a mixed-method process evaluation informed by the Reach, Effectiveness, Adoption, Implementation, Maintenance (RE-AIM) framework will be performed [54].

The reach is assessed by absolute numbers, proportions and representativeness of HCPs and patients participating, and by exploring reasons for non-participation. The effectiveness is determined by differences in the percentage of patients in whom deintensification of 1 or more cardiometabolic medications was (1) proposed and (2) implemented. Adoption is assessed by the evaluation of the training by HCPs, and the intended and reported use of training elements and tools in the CMR focused on deprescribing cardiometabolic medication. For implementation, the number of HCPs attending various parts of the training, the level of reflection and feedback on the consultations, and the conduct of the CMRs in accordance with the trained communication elements will be reported. For the latter, video recordings of consultations will be assessed using the codebook for rating clinical communication skills based on the Calgary-Cambridge Guide [55] and the Observer OPTION-5 [56] for shared decision making. For maintenance, HCPs are asked about the feasibility and willingness to implement CMRs focused on deprescribing cardiometabolic medication in daily practice. Patients are asked if they would like to receive similar counseling in the future.

The following data will be collected:

1. Participating HCPs in the intervention group will receive an online evaluation questionnaire to assess the training in terms of usefulness and intention to implement and use the various elements and tools.
2. For each participating patient, 1 member of the HCP team will document data for each of the following steps that make up a CMR:

- conduct of initial patient consultation.
- preparation and conduct of the pharmacotherapeutic analysis.
- drafting/exchanging a treatment plan by the HCPs.
- finalizing the treatment plan with the patient, including implemented medication changes.
- conduct of follow-up monitoring.

These data include the type of patient consultation (if applicable) distinguishing between face-to-face and telephone consultations, whether the consultation was videorecorded, and potential drug-related problems identified in the pharmacotherapeutic analysis. In addition, for a sample of 2 patients per participating HCP team, the time needed for each part of the CMR will be documented.

3. For each participating HCP team a structured time registration form will be completed for completing the training components.
4. Semi-structured interviews will be held with the HCPs intervention teams after follow-up data collection to assess barriers and facilitators for implementing the trained aspects in daily practice. The topic list for the semi-structured interview will be based on the framework from Grant et al. [57], and on earlier work from Baas et al. [27].
5. For each HCP intervention team, 2 patient consultations will be video- or audio-recorded. HCPs are instructed how to record these consultations in accordance with the national privacy legislation and request permission from the patient.

The process evaluation parameters will be described at HCP and patient level and tabulated per outcome. Where relevant, frequencies and percentages will be presented for the total group and according to subgroups of HCPs, i.e., pharmacists, general practitioners, support staff. The interviews with HCPs will be audio recorded, transcribed and thematically coded. Qualitative data will be illustrated with quotes.

### Economic evaluation

The cost-effectiveness of the intervention compared to care as usual will be assessed from the perspectives of the HCP, the healthcare payer, and society (see Additional file 4). Changes in health-related quality of life as measured by the EQ-5D-5L are expected to be small, given the aim of the intervention and the relatively short duration of the follow up. Therefore, next to costs per quality-adjusted life-year gained, costs per percentage point of deintensification and costs per reduction in the number of health/medication-related complaints with impact will be calculated. Full details on measurement and valuation of costs and health benefits, as well the planned analyses can be found in Additional file 4. In brief, resource use related to delivering and receiving the intervention, as well as cardiometabolic medication changes related to the intervention will be collected and valued at standard prices [58]. Quality-adjusted life-year will be calculated from EQ-5D-5L measurements, using the Dutch tariff [59] and linear interpolation between measurement points. Multiple imputation will be used to account for missing values and bootstrapping for analyzing uncertainty as per the Dutch guidelines [58]. If needed, a correction for baseline imbalances between groups will be made. The budget impact analysis will follow the Dutch guidelines [58] and use the dedicated tools from ZonMw [60] to provide insight into the total costs involved in implementation over a 5-year time horizon on a nation-wide scale using 3 scenarios concerning uptake (see Additional file 4).

### Patient involvement

For preparation of the research protocol, a member from the National Coalition of Dutch Patients (Patiëntenfederatie Nederland) has been consulted. Next, 2 patient representatives from the Dutch diabetes patient organization (Diabetesvereniging Nederland) and the Dutch association for people with cardiovascular diseases (Harteraad), respectively, have been involved in the preparations and will be involved in the conduct of the study. They provide input on study materials (i.e., patient information about participating, the CO-DEPRESCRIBE patient leaflet, the CO-DEPRESCRIBE conversation aids for HCPs), as well as the methods for collecting patient-reported data. The main results will be disseminated to study participants and other people living with cardiometabolic diseases and patient representatives will be involved in deciding on appropriate methods of and material for dissemination.

## Discussion

The CO-DEPRESCRIBE intervention aims to enable HCPs to initiate and conduct constructive consultations with patients of 75 years and older about deprescribing of cardiometabolic medication. The training focusses on knowledge and skills needed to discuss potential benefits and risks of deprescribing cardiometabolic medication, taking into account a patient’s personal preferences, goals, attitudes, and experiences regarding their cardiometabolic treatment. Training and tools are provided for involving patients, which are expected to improve the conduct of deprescribing conversations, and facilitate implementation into daily practice [61–63]. HCPs differ in their approach to eliciting patients’ preferences and in how they incorporate this in their decision making, and may need additional support to achieve patient-centered care and involve patient in shared decision making for deprescribing [13, 64, 65]. It is hypothesized that the training program will lead to more proactive and patient-centered deprescribing of cardiometabolic medication. It is expected that more patient-centered communication and shared decision making in conversations about possibilities for deprescribing cardiometabolic medication will increase the likelihood to deintensify such medication during a CMR [26, 66, 67].

Strengths of this study include its close alignment with current practice in primary care, involvement of multidisciplinary HCP teams, and the modularity of the intervention, which allows HCPs to use components as needed. The need for incorporation of interventions into existing care processes to foster the implementation of deprescribing has already been pointed out [68]. Moreover, the intervention is based on a combination of previously developed and tested components, and aligns with recently developed frameworks for deprescribing interventions [69–71]. A cluster-randomized trial is considered optimal to account for clustering of patients within HCP teams. By comprehensively evaluating the intervention by means of a mixed-methods process evaluation at HCP and patient level and conducting an economic evaluation alongside to the effect evaluation including a range of clinical and patient-reported outcomes, it is expected to fill gaps in knowledge needed for sustainable implementation of deprescribing [72, 73].

Potential limitations of this study include that HCPs in the control group might be prompted to focus more on deprescribing cardiometabolic medications by participating in the study. Similarly, patients in the control group might be prompted to carefully consider deprescribing and proactively engage in discussing possibilities for deprescribing due to participating in the study [74]. Furthermore, there is debate about the core outcomes for deprescribing research [75, 76]. Our primary outcome, the percentage of patients with at least 1 cardiometabolic medication deintensified, is considered an important outcome for implementation interventions but does not necessarily reflect optimal treatment for all patients. By including secondary outcomes and conducting subgroup analysis, the impact of this limitation will be explored. The study, however, is not powered and the follow-up is too short for any formal testing of safety and clinical outcomes of the intervention. Finally, there is a risk that the most frail patients in need of deprescribing will not participate or may not complete participation. Background characteristics, reasons for not participating and data on loss to follow-up will be collected to gain insight in potential bias as well as representativeness of our final study population.

In summary, by assessing the effects of a carefully designed training program for primary care HCP teams, this study will contribute to the emerging evidence on how to successfully implement a complex treatment intervention, that is, deprescribing of cardiometabolic medications in older patients.

### Electronic supplementary material

Below is the link to the electronic supplementary material.

Supplementary Material 1

Supplementary Material 2

Supplementary Material ﻿3

Supplementary Material 4

## Data Availability

No datasets were generated or analysed during the current study.

## Abbreviations

CAT-Pharm: Communication Assessment Tool for the Pharmacy setting
CMR: Clinical medication review
CO-DEPRESCRIBE: Communication training to involve Older people in decisions to DEPRESCRIBE
GP: General practitioner
HCP: Healthcare provider
iMTA MCQ: Institute for Medical Technology Assessment Medical Consumption Questionnaire
PREM: Patient Reported Experience Measure
RE-AIM: Reach, Effectiveness, Adoption, Implementation, Maintenance
rPATD: Revised Patients Attitudes Towards Deprescribing
SDM-Q-9: Shared Decision Making Questionnaire
TFI: Tilburg Frailty Indicator

## References

[1] Nederlands Huisartsen Genootschap i.s.m. andere beroepsorganisaties/instanties/ verenigingen [Dutch College of General Practitioners in collaboration with other professional organisations].Module Minderen En Stoppen, Onderdeel van de Multidisciplinaire Richtlijn Polyfarmacie Bij Ouderen [Module Deprescribing, part of the Multidisciplinary Guideline Polypharmacy in Elderly]. 2020; (December). https://richtlijnen.nhg.org//files/2020-11/Final_Module%20Minderen%20en%20stoppen%20van%20medicatie.pdf.

[2] Farrell B, Black C, Thompson W (2017). Deprescribing antihyperglycemic agents in older persons: evidence-based clinical practice guideline. Can Fam Physician.

[3] Scott IA, Hilmer SN, Reeve E (2015). Reducing inappropriate polypharmacy: the process of deprescribing. JAMA Intern Med.

[4] Reeve E, Gnjidic D, Long J, Hilmer S (2015). A systematic review of the emerging definition of ‘deprescribing’ with network analysis: implications for future research and clinical practice. Br J Clin Pharmacol.

[5] Christiaens A, Henrard S, Sinclair AJ, Tubach F, Bonnet-Zamponi D, Zerah L (2023). Deprescribing glucose-lowering therapy in older adults with diabetes: a systematic review of recommendations. J Am Med Dir Assoc.

[6] Steinman MA, Landefeld CS (2018). Overcoming Inertia to Improve Medication Use and Deprescribing. JAMA - J Am Med Assoc.

[7] Morehead S. A Guide to Deprescribing Antihypertensives. Primary Health Tasmania. 2022; (December). https://www.primaryhealthtas.com.au/wp-content/uploads/2023/03/A-guide-to-deprescribing-antihypertensives.pdf.

[8] Hart HE, Ditzel K, Rutten GE (2019). De-intensification of blood glucose lowering medication in people identified as being over-treated: a mixed methods study. Patient Prefer Adherence.

[9] Oktora MP, Kerr KP, Hak E, Denig P (2021). Rates, determinants and success of implementing deprescribing in people with type 2 diabetes: a scoping review. Diabet Med.

[10] Pilla SJ, Jalalzai R, Tang O (2023). A national physician survey of deintensifying diabetes medications for older adults with type 2 diabetes. Diabetes Care.

[11] Brunner L, Rodondi N, Aubert CE (2022). Barriers and facilitators to deprescribing of cardiovascular medications: a systematic review. BMJ Open.

[12] Crutzen S, Baas G, Abou J (2020). Barriers and enablers of older patients to deprescribing of cardiometabolic medication: a focus group study. Front Pharmacol.

[13] Abou J, Crutzen S, Tromp V (2022). Barriers and enablers of healthcare providers to deprescribe cardiometabolic medication in older patients: a focus group study. Drugs Aging.

[14] Seewoodharry M, Khunti K, Davies MJ, Gillies C, Seidu S (2022). Attitudes of older adults and their carers towards de-prescribing: a systematic review. Diabet Med.

[15] Reeve E, Shakib S, Hendrix I, Roberts MS, Wiese MD (2014). Review of deprescribing processes and development of an evidence-based, patient-centred deprescribing process. Br J Clin Pharmacol.

[16] Williams ME, Pulliam CC, Hunter R (2004). The short-term effect of interdisciplinary medication review on function and cost in ambulatory elderly people. J Am Geriatr Soc.

[17] McCarthy C, Clyne B, Boland F (2022). GP-delivered medication review of polypharmacy, deprescribing, and patient priorities in older people with multimorbidity in Irish primary care (SPPiRE Study): a cluster randomised controlled trial. PLoS Med.

[18] Lenander C, Elfsson B, Danielsson B, Midlöv P, Hasselström J (2014). Effects of a pharmacist-led structured medication review in primary care on drug-related problems and hospital admission rates: a randomized controlled trial. Scand J Prim Health Care.

[19] Jódar-Sánchez F, Malet-Larrea A, Martín JJ (2015). Cost-utility analysis of a medication review with follow-up service for older adults with polypharmacy in community pharmacies in Spain: the conSIGUE program. PharmacoEconomics.

[20] Romskaug R, Skovlund E, Straand J (2020). Effect of clinical geriatric assessments and collaborative medication reviews by geriatrician and family physician for improving health-related quality of life in home-dwelling older patients receiving polypharmacy: a cluster randomized clinical trial. JAMA Intern Med.

[21] Lee JQ, Ying K, Lun P (2020). Intervention elements to reduce inappropriate prescribing for older adults with multimorbidity receiving outpatient care: a scoping review. BMJ Open.

[22] Radcliffe E, Servin R, Cox N (2023). What makes a multidisciplinary medication review and deprescribing intervention for older people work well in primary care? A realist review and synthesis. BMC Geriatr.

[23] Bužančić I, Kummer I, Držaić M, Ortner Hadžiabdić M (2022). Community-based pharmacists’ role in deprescribing: a systematic review. Br J Clin Pharmacol.

[24] Raman-Wilms L, Farrell B, Sadowski C, Austin Z (2019). Deprescribing: an educational imperative. Res Soc Adm Pharm.

[25] Sawan M, Reeve E, Turner J (2020). A systems approach to identifying the challenges of implementing deprescribing in older adults across different health-care settings and countries: a narrative review. Expert Rev Clin Pharmacol.

[26] Crutzen S, Baas G, Denig P, Heringa M, Taxis K (2023). Pharmacist-led intervention aimed at deprescribing and appropriate use of cardiometabolic medication among people with type 2 diabetes. Res Soc Adm Pharm.

[27] Baas G, Crutzen S, Smits S, Denig P, Taxis K, Heringa M (2024). Process evaluation of a pharmacist-led intervention aimed at deprescribing and appropriate use of cardiometabolic medication among adult people with type 2 diabetes. Basic Clin Pharmacol Toxicol.

[28] Nederlands Huisartsen Genootschap i.s.m. andere beroepsorganisaties/instanties/verenigingen [Dutch College of General Practitioners in collaboration with other professional organisations]. Module Medicatiebeoordeling, Onderdeel van de Multidisciplinaire Richtlijn Polyfarmacie bij ouderen [Module Clinical Medication Review, part of Multidisciplinary Guideline Polypharmacy in Elderly]. 2019;(September). https://richtlijnen.nhg.org//files/2020-05/final_module_medicatiebeoordeling_2019.pdf.

[29] Greenhill N, Anderson C, Avery A, Pilnick A (2011). Analysis of pharmacist-patient communication using the Calgary-Cambridge guide. Patient Educ Couns.

[30] Elwyn G, Durand MA, Song J (2017). A three-talk model for shared decision making: multistage consultation process. BMJ.

[31] Stiggelbout AM, Pieterse AH, De Haes JCJM (2015). Shared decision making: concepts, evidence, and practice. Patient Educ Couns.

[32] Crutzen S, Abou J, Smits SE (2021). Older people’s attitudes towards deprescribing cardiometabolic medication. BMC Geriatr.

[33] Vervloet M, Lamboo A, Koster E, van Dijk L (2018). Betere Baliegesprekken met COM-MA-training: aansluiten bij behoeften en voorkeuren van patiënten. [Better consultation with COM-MA training: tailor to needs and preferences of patients]. Pharm Weekbl.

[34] Fried TR, Tinetti ME, Iannone L, O’Leary JR, Towle V, Van Ness PH (2011). Health outcome prioritization as a tool for decision making among older persons with multiple chronic conditions. Arch Intern Med.

[35] Olesen JB, Torp-Pedersen C, Hansen ML, Lip GYH (2012). The value of the CHA 2DS 2-VASc score for refining stroke risk stratification in patients with atrial fibrillation with a CHADS 2 score 0–1: a nationwide cohort study. Thromb Haemost.

[36] Lip GYH, Frison L, Halperin JL, Lane DA (2011). Comparative validation of a novel risk score for predicting bleeding risk in anticoagulated patients with atrial fibrillation: the HAS-BLED (hypertension, abnormal renal/liver function, stroke, bleeding history or predisposition, labile inr, elderly, drugs/alcohol concomitantly) score. J Am Coll Cardiol.

[37] O’Brien EC, Simon DN, Thomas LE (2015). The ORBIT bleeding score: a simple bedside score to assess bleeding risk in atrial fibrillation. Eur Heart J.

[38] World Health Organization. ATC/DDD Index. 2023. Available from. https://www.whocc.no/atc_ddd_index/. Accessed April 3, 2024.

[39] Verdoorn S, Kwint HF, Blom J, Gussekloo J, Bouvy ML (2018). DREAMeR: drug use reconsidered in the Elderly using goal attainment scales during Medication Review; study protocol of a randomised controlled trial. BMC Geriatr.

[40] Reeve E, Low LF, Shakib S, Hilmer SN (2016). Development and validation of the revised patients’ attitudes towards deprescribing (rPATD) questionnaire: versions for older adults and caregivers. Drugs Aging.

[41] George J, Phun YT, Bailey MJ, Kong DCM, Stewart K. Development and Validation of the Medication Regimen Complexity Index. *Ann Pharmacother*. 2004;38(9):1369-76. 2004;38(9):1369-76. 10.1345/aph.1D479 15266038

[42] de Vries ST, Keers JC, Visser R (2014). Medication beliefs, treatment complexity, and non-adherence to different drug classes in patients with type 2 diabetes. J Psychosom Res.

[43] Rodenburg-Vandenbussche S, Pieterse AH, Kroonenberg PM (2015). Dutch translation and psychometric testing of the 9-item shared decision making questionnaire (SDM-Q-9) and shared decision making questionnaire-physician version (SDM-Q-Doc) in primary and secondary care. PLoS ONE.

[44] Scala D, Mucherino S, Wirth F (2022). Developing and piloting a communication assessment tool assessing patient perspectives on communication with pharmacists (CAT-Pharm). Int J Clin Pharm.

[45] Herdman M, Gudex C, Lloyd A (2011). Development and preliminary testing of the new five-level version of EQ-5D (EQ-5D-5L). Qual Life Res.

[46] iMTA Productivity and Health Research Group (2018). Manual iMTA Medical cost questionnaire (iMCQ).

[47] Gobbens RJJ, van Assen MALM, Luijkx KG, Wijnen-Sponselee MT, Schols JMGA (2010). The Tilburg Frailty Indicator: Psychometric Properties. J Am Med Dir Assoc.

[48] Fransen MP, Van Schaik TM, Twickler TB, Essink-Bot ML (2011). Applicability of internationally available health literacy measures in the Netherlands. J Health Commun.

[49] Duncan P, Murphy M, Man MS, Chaplin K, Gaunt D, Salisbury C (2018). Development and validation of the Multimorbidity Treatment Burden Questionnaire (MTBQ). BMJ Open.

[50] Vervloet M, van Dijk L, Rademakers JJDJM (2018). Recognizing and addressing limited PHarmaceutical literacy: development of the RALPH interview guide. Res Soc Adm Pharm.

[51] Harris PA, Taylor R, Thielke R, Payne J, Gonzalez N, Conde JG (2009). Research electronic data capture (REDCap)--a metadata-driven methodology and workflow process for providing translational research informatics support. J Biomed Inf.

[52] Harris PA, Taylor R, Minor BL et al. The REDCap consortium: building an international community of software platform partners. J Biomed Inf. 2019;95. 10.1016/j.jbi.2019.103208 PMC7254481 31078660

[53] Nederlands Huisartsen Genootschap [Dutch College of General Practitioners]. International Classification of Primary Care version 10. 2022; (April). https://viewers.nhg.org/icpcviewer/. Accessed April 3, 2024.

[54] Glasgow RE, Harden SM, Gaglio B (2019). RE-AIM planning and evaluation framework: adapting to new science and practice with a 20-year review. Front Public Heal.

[55] Iversen ED, Wolderslund MO, Kofoed PE (2020). Codebook for rating clinical communication skills based on the Calgary-Cambridge Guide. BMC Med Educ.

[56] Barr PJ, O’Malley AJ, Tsulukidze M, Gionfriddo MR, Montori V, Elwyn G (2015). The psychometric properties of Observer OPTION5, an observer measure of shared decision making. Patient Educ Couns.

[57] Grant A, Treweek S, Dreischulte T, Foy R, Guthrie B (2013). Process evaluations for cluster-randomised trials of complex interventions: a proposed framework for design and reporting. Trials.

[58] Zorginstituut Nederland [National Health Care Institute]. Richtlijn Voor Het Uitvoeren van Economische Evaluaties in de Gezondheidzorg. 2024; (January). https://www.zorginstituutnederland.nl/over-ons/publicaties/publicatie/2024/01/16/richtlijn-voor-het-uitvoeren-van-economische-evaluaties-in-de-gezondheidszorg. Accessed April 3, 2024.

[59] Versteegh M, Vermeulen M, Evers KMAA, de Wit S, Prenger GA, Stolk RA (2016). Dutch tariff for the five-level version of EQ-5D. Value Heal.

[60] Budget Impact Analyses in de praktijk. ZonMw. https://www.zonmw.nl/nl/artikel/budget-impact-analyse-bia. Accessed April 3, 2024.

[61] Turner JP, Richard C, Lussier MT (2018). Deprescribing conversations: a closer look at prescriber–patient communication. Ther Adv Drug Saf.

[62] Martin P, Tamblyn R, Benedetti A, Ahmed S, Tannenbaum C (2018). Effect of a pharmacist-led Educational intervention on inappropriate medication prescriptions in older adults: the D-PRESCRIBE Randomized Clinical Trial. J Am Med Assoc.

[63] Trenaman S, Willison M, Robinson B, Andrew M (2020). A collaborative intervention for deprescribing: the role of stakeholder and patient engagement. Res Soc Adm Pharm.

[64] Bayliss EA, Bayliss EA, Shetterly SM (2020). The OPTIMIZE patient- and family-centered, primary care-based deprescribing intervention for older adults with dementia or mild cognitive impairment and multiple chronic conditions: study protocol for a pragmatic cluster randomized controlled trial. Trials.

[65] Weir KR, Naganathan V, Carter SM (2021). The role of older patients’ goals in GP decision-making about medicines: a qualitative study. BMC Fam Pract.

[66] Zechmann S, Senn O, Valeri F (2020). Effect of a patient-centred deprescribing procedure in older multimorbid patients in Swiss primary care - A cluster-randomised clinical trial. BMC Geriatr.

[67] Anderson TS, Goyal P, Marcum ZA (2020). Implementing a proactive Deprescribing Approach to prevent adverse drug events. J Gen Intern Med.

[68] Wang J, Shen JY, Conwell Y (2024). Implementation considerations of deprescribing interventions: a scoping review. J Intern Med.

[69] Armistead LT, Sanders KA, Larson CK, Busby-Whitehead J, Ferreri SP (2022). A-TAPER: a framework for deprescribing medications effectively. Res Soc Adm Pharm.

[70] Mangin D, Lamarche L, Templeton JA (2023). Theoretical underpinnings of a model to reduce polypharmacy and its negative health effects: introducing the Team Approach to Polypharmacy evaluation and reduction (TAPER). Drugs Aging.

[71] Farrell B, Raman-Wilms L, Sadowski CA (2023). A proposed Curricular Framework for an Interprofessional Approach to Deprescribing. Med Sci Educ.

[72] Fellenor J, Britten N, Courtenay M (2021). A multi-stakeholder approach to the co-production of the research agenda for medicines optimisation. BMC Health Serv Res.

[73] Lundby C, Thompson W (2024). Advancing deprescribing: Learnings from the first international conference on deprescribing. Basic Clin Pharmacol Toxicol.

[74] McCarney R, Warner J, Iliffe S, Van Haselen R, Griffin M, Fisher P (2007). The Hawthorne Effect: a randomised, controlled trial. BMC Med Res Methodol.

[75] Martin-Kerry J, Taylor J, Scott S (2022). Developing a core outcome set for hospital deprescribing trials for older people under the care of a geriatrician. Age Ageing.

[76] van Poelgeest E, Seppala L, Bahat G (2023). Optimizing pharmacotherapy and deprescribing strategies in older adults living with multimorbidity and polypharmacy: EuGMS SIG on pharmacology position paper. Eur Geriatr Med 2023.

