# Supplementary material for: Effects of a multicomponent communication training to involve older people in decisions to DEPRESCRIBE cardiometabolic medication in primary care (CO-DEPRESCRIBE): protocol for a cluster randomized controlled trial with embedded process and economic evaluation
Source: BMC Prim Care. 2024 Jun 11;25:210. doi: 10.1186/s12875-024-02465-7 (PMC11165805; doi:10.1186/s12875-024-02465-7)
Supplement: Supplementary file 1 — Supplementary Material 1 [file 12875_2024_2465_MOESM1_ESM.docx]

# **Additional file 1: Comparison between previously evaluated pharmacist-led intervention and the CO-DEPRESCRIBE intervention**

| **Aspect** | **Previously evaluated pharmacist-led intervention^1,2^** | **CO-DEPRESCRIBE intervention** |
| --- | --- | --- |
| Scope | Deprescribing of cardiometabolic medication in ambulatory patients with type 2 diabetes and a high hypoglycemia risk in Dutch primary care | Proactive deprescribing of cardiometabolic medication in ambulatory patients of at least 75 years in Dutch primary care eligible for CMR |
| Brief description of intervention (estimated time investment for receiving training) | Face-to-face group training focusing on user problems, patient counselling, and recommendations for deprescribing cardiometabolic medication (6 hours), followed by performing tailored CMRs focusing on deprescribing cardiometabolic medication | Blended (online, face-to-face group and individual feedback) training program, focusing on patient-centered communication, shared decision making and recommendations for deprescribing cardiometabolic medication (11.5 hours), followed by performing tailored CMRs focusing on deprescribing cardiometabolic medication |
| Tools provided (as part of the intervention) | 1. A conversation aid on user problems, attitudes towards deprescribing and hypoglycemia 2. An agreement card for noting down treatment changes 3. Summary of guidelines for deprescribing cardiometabolic medication | 1. Patient leaflet for preparing patients 2. Graphical placemat for conversation navigating 3. Conversation aid#1 on collecting information on preferences, treatment goals, attitudes towards deprescribing 4. Conversation aid#2 on the different steps of shared decision making 5. Outcome Prioritization Tool 6. Several risk calculators for weighing potential benefits and risks of continuing or discontinuing medication |

CMRs = clinical medication reviews; HCP = healthcare provider

**References**

1. Crutzen S, Baas G, Denig P, Heringa M, Taxis K. Pharmacist-led intervention aimed at deprescribing and appropriate use of cardiometabolic medication among people with type 2 diabetes. *Res Soc Adm Pharm*. 2023;19(5):783-792.

2. Baas G, Crutzen S, Smits S, Denig P, Taxis K, Heringa M. Process evaluation of a pharmacist-led intervention aimed at deprescribing and appropriate use of cardiometabolic medication among adult people with type 2 diabetes. *Basic Clin Pharmacol Toxicol*. 2024 Jan;134(1):83-96.
