# Supplementary material for: Effects of a multicomponent communication training to involve older people in decisions to DEPRESCRIBE cardiometabolic medication in primary care (CO-DEPRESCRIBE): protocol for a cluster randomized controlled trial with embedded process and economic evaluation
Source: BMC Prim Care. 2024 Jun 11;25:210. doi: 10.1186/s12875-024-02465-7 (PMC11165805; doi:10.1186/s12875-024-02465-7)
Supplement: Supplementary file 2 — Supplementary Material 2 [file 12875_2024_2465_MOESM2_ESM.docx]

# **Additional file 2. A detailed description of the content of the different training modules**

| Module | Description of content |
| --- | --- |
| M**odule 1:**  e-learning with focus on Dutch multidisciplinary guideline module for “Deprescribing medication” | This e-learning consists of four short web lectures. The first web lecture covers general aspects of the Dutch multidisciplinary guideline module for deprescribing (1). After the lecture, questions are posed about which barriers and facilitators of deprescribing the trainee recognizes in their daily practice (2,3,4). The second web lecture is about which patients might benefit from deprescribing, and how deprescribing can be embedded in routine care activities. The six step cycle for deprescribing as depicted in the Dutch guideline module for deprescribing is introduced. The first step includes assessing the clinical condition of the patient, collecting information on the patient’s attitudes towards and experiences with chronic medication use, and discussing personal goals, preferences and expectations regarding health and medication use (5). Then a patient case is introduced, and the trainee is asked to assess if the patient is considered frail and/or a good candidate for deprescribing. The third web lecture covers the second step - analyzing medication use and checking for drug-related problems- and third step -considering possibilities for deprescribing- of the and lists instruments (e.g., the STRIP tool and Dutch STOPP criteria) that can help in evaluating the need for deprescribing (6, 7). The fourth web lecture covers the fourth -prioritizing and shared decision making with the patient-, fifth -composing a stepwise deprescribing plan- and sixth step -evaluating, monitoring and supporting- of the cycle and lists instruments (e.g., OPT and U-Prevent) that can support shared decision making (8, 9). After this lecture, another patient case is introduced, and the trainee is asked to analyze the patient’s medication by applying one (or more) of the instruments mentioned in the third lecture. |
| **Module 2:**  e-learning with focus on how to talk about possibilities for deprescribing | This e-learning consists of three short web lectures about patient-centered communication according to the Calgary-Cambridge consultation model, constructively discussing possibilities to deprescribe and how to apply the principles of shared decision making in a consultation in which changes in the treatment plan are discussed with the patient (10-12). After the first lecture, the trainee is asked to reflect upon recently conducted consultations, to see to which extent the trainee has or has not applied patient-centered communication. After the second lecture, a patient case is introduced. The trainee is asked to list potential reasons why the patient would like or dislike to have any of his diabetes medication deprescribed. After the third lecture, the trainee is asked what principles of shared decision making he/she applied during previous consultations in which different options were discussed with a patient. The trainee is also asked about new insights about shared decision making gained from watching the lecture, and if there are any aspects that the trainee would like to discuss or practice during the upcoming face-to-face training sessions. |
| **Module 3.1:**  face-to-face group training with focus on applying deprescribing guidelines in practice | The location for the first face-to-face small group training session is chosen based on the convenience of all participants and their respective locations. First, an interactive Wooclap-based multiple-choice quiz is held, to both test and freshen up knowledge about cardiometabolic-specific deprescribing guidelines (around 1 hour and 45 minutes). An example of a question is “For how long does a statin need to be ceased before evaluating muscle pain complaints?”. After each question, the answer is briefly discussed. Then for antihypertensives, antihyperglycemics, statins, antiplatelet agents and anticoagulants, the deprescribing guidelines are centrally discussed and summarized by the trainers. Next, the patient case from the e-learning is re-introduced. Trainees in groups of 2-4 persons are given 20 minutes to analyze the case in terms of appropriateness of both current cardiovascular and diabetes treatment, potential drug-related problems, and which explicit changes in medication they would propose, including SMART-goals. This is followed by a plenary discussion of the patient case, informed by six steps as proposed by the Dutch guidelines (13). |
| M**odule 3.2:** face-to-face group training with focus on collecting information on a patient’s preferences, goals, attitudes, and experiences | During the last one and a half hour of the first group session, the trainer recalls or activates prior knowledge about what characterizes a good patient consultation. After trainees are reminded about how they reflected upon recently conducted patient consultations in e-learning (second training module), they are asked to discuss their personal pitfalls in patient consultations with their neighboring trainees for 5 minutes before common pitfalls are plenary discussed. Then the trainer explains about patient typologies in relation to different (medication-specific) attitudes towards deprescribing, and how this might relate to different information needs of the patient (3, 14). The trainer elaborates on the importance of collecting information on the patient’s personal attitudes, preferences, experiences and goals relating to his/her pharmacotherapeutic treatment (introduced in the e-learning), as this should enable the trainee to carefully consider potential benefits and risks of deprescribing a certain drug for that specific patient. She also demonstrates the application of reflective listening; a technique that can elicit a more detailed explanation from the patient about the origin of a certain attitude or preference. Next, trainees perform a roleplaying exercise in pairs. While one trainee acts out a provided patient case, the other trainee conducts an initial consultation (in his/her role as HCP). Later, the roles are reversed, with the HCP of the first round now acting out a different patient case. After the exercise, the trainers present a conversation aid for collecting information on personal attitudes, preferences, experiences and goals on about deprescribing cardiometabolic medication during initial consultations. Then, trainees are given the assignment to conduct clinical medication reviews focused on discussing possibilities to deprescribe cardiometabolic medication and video-record themselves after the patient grants permission. To prepare for the second face-to-face training session, they are asked to reflect upon what went well and what could be improved in gaining insight into the patient’s attitudes and preferences about deprescribing cardiometabolic medication, and perform a pharmacotherapeutic analysis and note down their considerations, which tools they used and any difficulties they encountered. To conclude, the trainers discuss organizational barriers and facilitators for the successful implementation of deprescribing in primary care, and explain how a multidisciplinary approach is a key facilitator (4). Trainees are invited to think about what actions and/or agreements are needed locally to establish good communication and collaboration between the different HCPs. At the end, trainees complete a digital short evaluation form about the session. |
| **Module 4.1:**  face-to-face group training with focus on applying tools and addressing experienced barriers and facilitators | The location for the second face-to-face small group training session is chosen based on the convenience of all participants and their respective locations. The first part starts with briefly discussing which tools trainees used during the preparatory assignment that was given at the end of the previous training session (see module 3.2) to assess potential benefits and risks of deprescribing medication. Then the trainers elaborate on how U-Prevent calculators (introduced in the e-learning) can be used to quantify the potential benefit of continuing versus deintensifying cardiometabolic treatment terms of cardiovascular disease-free years, 10 years risk and/or lifetime risk (9). During an exercise, groups of 2-4 trainees receive two paper-based patient cases for whom they have to calculate lifetime risk and discuss the findings to practice the application of U-Prevent. During the plenary discussion, the trainers show for both patient cases how stopping a statin, allowing a less stringent HbA1c level, changing systolic blood pressure or stopping an antiplatelet agent would affect the lifetime risk. Then the trainers explain that HCPs need to consider the potential negative effects themselves, as the calculators only present the potential benefit of (an intensified) treatment. It is also discussed how to translate the risk scores to information that is usable for a patient in considering different options. Next, the trainers present the CHA2DS2–VASc for calculating the 1-year risk of a CVA, and the HASBLED and ORBIT to calculate the 1-year major bleeding risk (15-17). Again trainees perform an exercise in which they have to calculate both CVA and bleeding risks for two paper-based patient cases. In discussing the exercise, trainers relate the patient cases and the calculated risk scores to the guidelines on deprescribing antiplatelet agents and anticoagulants. To conclude this part, trainees’ experiences with the preparatory assignment are extensively discussed focusing on barriers and facilitators they experience. First in groups of 2-4 trainees for about 15 minutes, then in a 15 minute plenary session. Topics for the discussions include what drug-related problems have been analyzed, which tools have been used, what difficulties were encountered and how were these dealt with? |
| **Module 4.2:**  face-to-face group training with focus on practicing with shared decision making in the context of deprescribing | During the second part of the second group session, the trainers shortly look back on practicing on how to collect information on the patient’s attitudes towards and experiences with chronic medication use during the first face-to-face training session. The six step cycle for deprescribing that was introduced in the e-learning is revisited where shared decision making with the patient about prioritizing drugs to deprescribe and how to deprescribe cardiometabolic medication follows after exploring the patient’s attitudes and experiences related to their medication use, analyzing the patient’s medication use and considering possibilities for deprescribing (13). After summarizing evidence on the effects of shared decision making on patient involvement, satisfaction and assertiveness, the trainers show the steps of shared decision making as proposed by Stiggelbout’s model, and present a second conversation aid for practicing shared decision making based on this model (12). This model is a refinement of Elwyn’s three-talk model for shared decision making (11). Next, trainees receive one of two patient case descriptions with the outcomes of an initial patient consultation about the patient’s attitudes towards, preferences for and concerns if deprescribing cardiometabolic medication. As first exercise, pairs of trainees receiving the same case are asked to prepare for a follow-up patient consultation, in which they need to discuss the different deprescribing options with their patient. After preparation time, the patient consultations are acted out in plenary, where a professional actress plays the role of the two patient cases and the trainees take turns in leading the consultation. During the consultation, the patient actress moves between three labeled chairs to indicate when she feels resistant, neutral or willing to accept a proposal for a certain drug being deprescribed. The trainers explain how a patient showing resistance can be considered an opportunity to identify a certain barrier that the patient might experience, which can be addressed after identification.  After a brief recap of how reflective listening can elicit a more detailed explanation from the patient about the origin of a certain attitude or preference (see module 3.2), the trainers introduce a second exercise with the actress playing the patient with whom a follow-up consultation on discussing deprescribing options is planned. Trainees are divided in three groups and given 10 minutes to prepare focusing on specific steps of Stiggelbout’s shared decision making model. In the plenary setting, between one to three trainees conduct these steps. Other trainees were assigned to observe and give feedback. When discussing the exercise, the trainers give special attention to the tools, that is, how the two conversation aids, the U-Prevent calculators, and the patient leaflets on stopping specific medications can be used. Finally, trainees receive instructions for the final training module 5, for which they need to continue making video-recordings of themselves during patient consultations focusing on discussing possibilities to deprescribe cardiometabolic medication. At the end, trainees complete a digital short evaluation form about the session. |
| **Module** **5:** online feedback with focus on five domains of consultation | For the final online module, the trainee has access to a maximum of two of his/her own video-recorded consultations on a digital and secured platform, which is designed for receiving online feedback (18). The trainee first reviews the own consultations, and reflects on what went well and what could be improved for five different domains: I. opening the consultation, II. collecting information on the patient’s personal situation, wishes and attitudes regarding, III. explaining and shared decision-making, IV. closing the consultation and V. structure, communication style and building a relationship with the patient. Then, feedback is given by a trained observer on what went well and what could be improved for the same five domains. The different aspects in domains I, IV and V are based on the Calgary-Cambridge model (10), while the aspects in domains II and III are based on Stiggelbout’s shared decision making model (12). The trainee can also pose questions or respond to the feedback from the trainer on the same platform. |

**References**

1. Module Minderen en stoppen medicatie, Onderdeel van de Multidisciplinaire Richtlijn Polyfarmacie bij ouderen [Module Deprescribing, part of the Multidisciplinary Guideline Polypharmacy in Elderly]. 2020; (December). Available: https://richtlijnen.nhg.org//files/2020-11/Final_Module%20Minderen%20en%20stoppen%20van%20medicatie.pdf

2. Brunner L, Rodondi N, Aubert CE. Barriers and facilitators to deprescribing of cardiovascular medications: A systematic review. *BMJ Open*. 2022;12(12):e061686.

3. Crutzen S, Baas G, Abou J, et al. Barriers and enablers of older patients to deprescribing of cardiometabolic medication: a focus group study. *Front Pharmacol*. 2020;11:555327.

4. Abou J, Crutzen S, Tromp V, et al. Barriers and enablers of healthcare providers to deprescribe cardiometabolic medication in older patients: a focus group study. *Drugs and Aging*. 2022;39(3):209-221.

5. Module Medicatiebeoordeling, Onderdeel van de Multidisciplinaire Richtlijn Polyfarmacie bij ouderen [Module Clinical Medication Review, part of Multidisciplinary Guideline Polypharmacy in Elderly]. 2019;(September). Available: https://richtlijnen.nhg.org//files/2020-05/final_module_medicatiebeoordeling_2019.pdf

6. Drenth‐van Maanen AC, Leendertse AJ, Jansen PA et al. The Systematic Tool to Reduce Inappropriate Prescribing (STRIP): Combining implicit and explicit prescribing tools to improve appropriate prescribing. *J Eval Clin Pract*. 2018;24(2), 317-322.

7. Knol W, Verduijn MM, Lelie-Van der Zande ACAM, et al. Onjuist geneesmiddelgebruik bij ouderen opsporen. De herziene STOPP en START criteria. *Ned Tijdschr Geneeskd*.2015;159:A8904.

8. Stegmann ME, Festen S, Brandenbarg D, et al. Using the Outcome Prioritization Tool (OPT) to assess the preferences of older patients in clinical decision-making: A review.Maturitas. 2019 Oct;128:49-52.

9. U-prevent team. U-Prevent, powered by ORTEC. Available: <https://u-prevent.nl/calculators>

10. Greenhill N, Anderson C, Avery A, et al. Analysis of pharmacist-patient communication using the Calgary-Cambridge guide. *Patient Educ Couns*. 2011;83(3):423-431.

11. Elwyn G, Durand MA, Song J, et al. A three-talk model for shared decision making: Multistage consultation process. *BMJ*. 2017;359:4891.

12. Stiggelbout AM, Pieterse AH, De Haes JCJM. Shared decision making: Concepts, evidence, and practice. *Patient Educ Couns*. 2015;98(10):1172-1179.

13. Cyclus stoppen en minderen van medicatie, Onderdeel van de Multidisciplinaire Richtlijn Polyfarmacie bij ouderen [Cyclus Deprescribing, part of Multidisciplinary Guideline Polypharmacy in Elderly]. 2021. Available: https://richtlijnen.nhg.org//files/2021-02/210202%20Cyclus%20stoppen%20en%20minderen%20van%20medicatie.pdf

14. Weir K, Nickel B, Naganathan V, et al. Decision-making preferences and deprescribing: perspectives of older adults and companions about their medicines. J Gerontol B Psychol Sci Soc Sci. 2018;73(7):e98-e107.

15. Olesen JB, Torp-Pedersen C, Hansen ML, Lip GYH. The value of the CHA 2DS 2-VASc score for refining stroke risk stratification in patients with atrial fibrillation with a CHADS 2 score 0-1: A nationwide cohort study. Thromb Haemost. 2012;107(6):1172-1179.

16. Lip GYH, Frison L, Halperin JL, Lane DA. Comparative Validation of a Novel Risk Score for Predicting Bleeding Risk in Anticoagulated Patients With Atrial Fibrillation: The HAS-BLED (Hypertension, Abnormal Renal/Liver Function, Stroke, Bleeding History or Predisposition, Labile INR, Elderly, Drugs/Alcohol Concomitantly) Score. J Am Coll Cardiol. 2011;57(2):173-180.

17. O’Brien EC, Simon DN, Thomas LE, et al. The ORBIT bleeding score: a simple bedside score to assess bleeding risk in atrial fibrillation. Eur Heart J. 2015;36(46):3258-3264.

18. Vervloet M, Lamboo A, Koster E, et al. Betere baliegesprekken met COM-MA-training: aansluiten bij behoeften en voorkeuren van patiënten. Pharm Weekbl. 2018;153(10):16-17.
