# Supplementary material for: Effects of a multicomponent communication training to involve older people in decisions to DEPRESCRIBE cardiometabolic medication in primary care (CO-DEPRESCRIBE): protocol for a cluster randomized controlled trial with embedded process and economic evaluation
Source: BMC Prim Care. 2024 Jun 11;25:210. doi: 10.1186/s12875-024-02465-7 (PMC11165805; doi:10.1186/s12875-024-02465-7)
Supplement: Supplementary file 3 — Supplementary Material ﻿3 [file 12875_2024_2465_MOESM3_ESM.docx]

**Additional file 3. Schematic overview of data collection**

| **TIMEPOINT** | 365-0 days prior to initial consultation | 120-0 days prior to initial consultation | 14-0 days prior to initial consultation | 7-0 days prior to initial consultation | day 0: initial consultation and follow-up | 28-62 days after initial consultation | 175-203 days after initial consultation | 175-203 days after initial consultation | 122-242 days after initial consultation | 0-272 days after initial consultation |
| --- | --- | --- | --- | --- | --- | --- | --- | --- | --- | --- |
| **INTERVENTION** |  |  |  |  |  |  |  |  |  |  |
| *Clinical medication review* |  |  |  |  |  |  |  |  |  |  |
| **ASSESSMENTS** (number of items for questionnaires) |  |  |  |  |  |  |  |  |  |  |
| Medication data |  | X |  |  |  |  |  |  | X |  |
| Clinical data | X |  |  |  |  |  |  |  |  | X |
| Health/medication-related complaints with impact* (11) |  |  | X |  |  |  | X |  |  |  |
| EQ-5D-5L** (6) |  |  | X |  |  |  | X |  |  |  |
| rPATD – general* (12) |  |  | X |  |  |  | X |  |  |  |
| Health- and medication literacy (4) |  |  | X |  |  |  |  |  |  |  |
| Demographics (2) |  |  |  | X |  |  |  |  |  |  |
| TFI (15) |  |  |  | X |  |  |  |  |  |  |
| iMTA MCQ* (12) |  |  |  | X |  |  |  | X |  |  |
| rPATD – specific* (10) |  |  |  | X |  |  |  | X |  |  |
| SDM-Q-9* (9) |  |  |  |  |  | X |  |  |  |  |
| CAT-Pharm* (12) |  |  |  |  |  | X |  |  |  |  |
| PREM*** (4) |  |  |  |  |  | X |  |  |  |  |

CAT-Pharm = Communication Assessment Tool for the Pharmacy setting, iMTA MCQ = Institute for Medical Technology Assessment Medical Consumption Questionnaire, PREM = Patient Reported Experience Measure, rPATD = revised Patients Attitudes Towards Deprescribing, SDM-Q-9 = Shared Decision Making Questionnaire, TFI = Tilburg Frailty Indicator; * questionnaires with minor adaptation; ** health-related quality of life; *** only administered for patients in intervention group
