# Supplementary material for: Effects of a multicomponent communication training to involve older people in decisions to DEPRESCRIBE cardiometabolic medication in primary care (CO-DEPRESCRIBE): protocol for a cluster randomized controlled trial with embedded process and economic evaluation
Source: BMC Prim Care. 2024 Jun 11;25:210. doi: 10.1186/s12875-024-02465-7 (PMC11165805; doi:10.1186/s12875-024-02465-7)
Supplement: Supplementary file 4 — Supplementary Material 4 [file 12875_2024_2465_MOESM4_ESM.docx]

# **Additional file 4: Additional information on economic evaluation**

The cost-effectiveness of the intervention compared to care as usual will be assessed from the perspectives of the healthcare provider (HCP), the healthcare payer, and society (see Table below). Cost-effectiveness will be assessed for three outcomes: QALYs, percentage point of deintensification and number of health/medication-related complaints with impact. Results will be presented as incremental cost-effectiveness ratios (ICERs), cost-effectiveness planes and cost-effectiveness acceptability curves. When the difference in health benefits (i.e., QALYs, percentage points of deintensification, number of complaints) is both statistically not significant and clinically irrelevant, an ICER will not be calculated, to avoid irrelevant analyses. An additional budget impact analysis is conducted. We will follow the 2024 update of the Dutch guideline on economic evaluation and the references provided in this guideline^1^.

**Health benefits**

The scores on the EQ-5D-5L obtained at baseline and six months follow-up are transformed using the Dutch tariff^2^, before using linear interpolation between measurement points to calculate QALY gains related to the intervention by comparing the intervention and control group. Differences in percentage points of deintensification refers to the difference in the proportion of patients with at least one cardiometabolic medication deintensified (primary outcome) between intervention and control group. Health/medication-related complaints are measured by a self-report questionnaire, with two items inquiring about the presence (dichotomous) and impact (on a five point scale) for a number of complaints^3^. For this study, only items about complaints that could be related to cardiometabolic medication use are administered. Only complaints with moderate to severe impact are counted.

**Costs and valuations**

For the costs, change in healthcare consumption including medication will be measured next to intervention costs. We identify different cost elements with respective valuations for the different perspectives (see table below). All cost items will be valued and for each patient variables will be created that contain estimated costs for the full six month period. Changes in medication will be based on pharmacy dispensing data. Changes in other healthcare consumption will be primarily based on the Medical Consumption Questionnaire (iMTA MCQ) administered at baseline and at six months follow-up ^4^. This instrument measures the extent to which the patient has received different types of care in the previous three months. These data will be complemented with data on unplanned hospitalizations and emergency visits from the patients’ general practice records during follow-up. The valuation of cardiometabolic medication is based on the price information as recommended in the Dutch cost manual ^5^ (National Health Care Institute, medicijnkosten.nl).^6^ For valuation of reduced income for pharmacists due to medication that is discontinued after the intervention, we will account for differences in dispensing fees. For valuation of other healthcare resources, we will use the unit prices from the Dutch cost manual^5^. We will not include productivity losses or patient time costs, since we consider these negligible for this population at age 75 and over. Time or fee spent on participation in the training program is not included as intervention cost, as HCPs are required to participate in such education to maintain their professional registration.

***Table. Cost elements and respective valuations for the different perspectives***

| Perspective | Cost element | Valuation (*, multiplication sign) |
| --- | --- | --- |
| HCP | Reduced income due to cardiometabolic medication that is discontinued after intervention | Dispensing fee * the number of cardiometabolic medications discontinued |
| HCP | Providing a clinical medication review | Amount that is remunerated minus (number of hours spent by HCP * unit price HCP) |
| Payer | Cardiometabolic medication changes | Based on cardiometabolic medication changed in relation to deintensification, valued using price information from the National Health Care Institute |
| Payer | Remunerating of clinical medication review | Clinical medication review conducted, valued using tariffs |
| Payer | Self-reported healthcare consumption | Based on iMTA MCQ changes, valued using tariffs |
| Societal | Developing training program | Estimated costs for developing training |
| Societal | Receiving a clinical medication review | Patient travel costs |
| Societal | Clinical medication review | Time of HCPs for conducting the clinical medication review * unit price HCP |
| Societal | Cardiometabolic medication use | Based on cardiometabolic medication changed, valued at prices from medicijnkosten.nl, accounting for co-payments when relevant |
| Societal | Other healthcare consumption | Based on iMTA MCQ changes, valued using unit prices |

**Data handling and analyses**

Missing values will be imputed using MICE while attention will be paid to handling missings due to drop out. Baseline imbalances between intervention and control group will be checked and corrected for. Bootstrapping will be applied to generate a cost-effectiveness plane representing the uncertainty around the study results concerning costs and health benefits. A cost-effectiveness acceptability curve will be used to present the probability that the intervention is cost-effective for different threshold values. This is followed by a value of information analysis to help interpret these findings.

A budget impact analysis is conducted following the Dutch guidelines^1^ and using the dedicated tools from ZonMw^5^ to provide insight into the estimated costs involved for implementation over a 5-year time horizon on a nation-wide scale using the following three scenarios. Scenario 1 will be directly based on study findings, extrapolating these using information on the number of pharmacies and their clients in the Netherlands. Scenario 2 will adjust the estimates from scenario 1 for possible bias introduced due to participating HCPs being more active. That is, this scenario will apply reduced proportions of participation and deprescribing, using the lower boundaries of confidence intervals of our effect estimates. Scenario 3 will adjust the estimates of scenario 2 for capacity constraints in pharmacies. This is assumed to imply that only 75% of planned reviews can be performed.

**References**

1. Zorginstituut Nederland [National Health Care Institute]. Richtlijn Voor Het Uitvoeren van Economische Evaluaties in de Gezondheidzorg. 2024; (January). Available from: <https://www.zorginstituutnederland.nl/over-ons/publicaties/publicatie/2024/01/16/richtlijn-voor-het-uitvoeren-van-economische-evaluaties-in-de-gezondheidszorg>.

2. Versteegh M, M. Vermeulen K, M. A. A. Evers S, de Wit GA, Prenger R, A. Stolk E. Dutch Tariff for the Five-Level Version of EQ-5D. Value Heal. 2016;19(4):343-352

3. Verdoorn S, Kwint HF, Blom J, Gussekloo J, Bouvy ML. DREAMeR: Drug use Reconsidered in the Elderly using goal Attainment scales during Medication Review; Study protocol of a randomised controlled trial. BMC Geriatr. 2018;18(1):1-10

4. iMTA Productivity and Health Research Group. Manual iMTA Medical Cost Questionnaire (iMCQ). Rotterdam: iMTA, Erasmus University Rotterdam, 2018.

5. Hakkaart-van Roijen L, Van der Linden N, Bouwmans C, Kanters T, Tan SS. Kostenhandleiding. Methodologie van kostenonderzoek en referentieprijzen voor economische evaluaties in de gezondheidszorg. 2015;. Available from: [https://www.zorginstituutnederland.nl/binaries/zinl/documenten/publicatie/2016/02/29/richtlijn-voor-het-uitvoeren-van-economische-evaluaties-in-de-gezondheidszorg/Richtlijn%2Bvoor%2Bhet%2Buitvoeren%2Bvan%2Beconomische%2Bevaluaties%2Bin%2Bde%2Bgezondheids](https://www.zorginstituutnederland.nl/binaries/zinl/documenten/publicatie/2016/02/29/richtlijn-voor-het-uitvoeren-van-economische-evaluaties-in-de-gezondheidszorg/Richtlijn%2Bvoor%2Bhet%2Buitvoeren%2Bvan%2Beconomische%2Bevaluaties%2Bin%2Bde%2Bgezondheidszorg%2B%2528verdiepingsmodules%2529.pdf). Accessed December 12, 2023

6. Zorginstituut Nederland [National Health Care Institute]. Pharmacy Price Information. [www.medicijnkosten.nl](http://www.medicijnkosten.nl)
